# Relapse of Immune Thrombotic Thrombocytopenic Purpura Following Vaccination with COVID19 mRNA Vaccine

**DOI:** 10.1055/s-0041-1732342

**Published:** 2021-08-09

**Authors:** Katerina Pavenski

**Affiliations:** 1 Department of Medicine and Laboratory Medicine, St. Michael's Hospital – Unity Health Toronto, Toronto, Canada; 2 Departments of Medicine and Laboratory Medicine and Pathobiology, University of Toronto, Canada

**Keywords:** ADAMS/ADAMTS 13, thrombotic thrombocytopenic purpura (TTP / HUS), thrombocytopenia

## Abstract

An 84 year old male with a previous history of immune thrombotic thrombocytopenic purpura (iTTP) received the first dose of COVID19 mRNA vaccine (Pfizer-Biontech). Seven days later he was diagnosed with iTTP relapse. He received in-patient treatment with therapeutic plasma exchange, high dose steroids and rituximab and subsequently recovered. This case report highlights the need to monitor patients with iTTP following vaccination.

## Dear Editor

Immune TTP is a rare autoimmune disease that presents with acute fragmentation hemolytic anemia, thrombocytopenia and microvascular thrombosis and is defined by severe ADAMTS13 deficiency. 1 More recently iTTP was also recognized as a chronic disease, due to its high rate of relapse and chronic complications. 1 Vaccination, especially against viral pathogens, has been previously reported to be associated with TTP onset and/or relapse within 1–2 weeks. 2 Pathophysiology is unknown but may include immune dysregulation. 3 COVID19 vaccines have been associated with hematological complications, including thrombocytopenia with thrombosis. 4 Recently, two case reports described association between immune TTP and COVID19 vaccines, one following adenovirus vector vaccine 5 and one following mRNA vaccine. 3 I would like to share the second case report of immune TTP relapse, which occurred within 7 days of receiving the first dose of COVID19 mRNA vaccine. Patient consent was obtained and publication of this case report was approved by the IRB. This adverse event was reported to the local public health unit.

An 84 year old male presented for a scheduled follow-up appointment on Day 0. His medical history was significant for immune TTP, diagnosed and treated 14 months earlier. His first episode of TTP was complicated by multiple cerebral infarcts, myocardial infarction, and mild renal dysfunction. His nadir platelet count was 18 × 10 ^9^ /L, nadir hemoglobin 6.1 g/dL, and peak LDH was 922U/L (ULN 195). Admission ADAMTS13 activity was below 1 percent U/mL and inhibitor was more than 15, consistent with diagnosis of iTTP. He was treated with therapeutic plasma exchange (TPE), high dose steroids, rituximab and caplacizumab. The patient recovered without any long-term sequelae and remission ADAMTS13 activity was 45 percent, last measured 10 months prior. His other medical history included remote treated prostate cancer, hypertension, type 2 diabetes, gout, and hypercholesterolemia. Following discharge, he was able to return to his physically active and independent living.

**Fig. 1:**
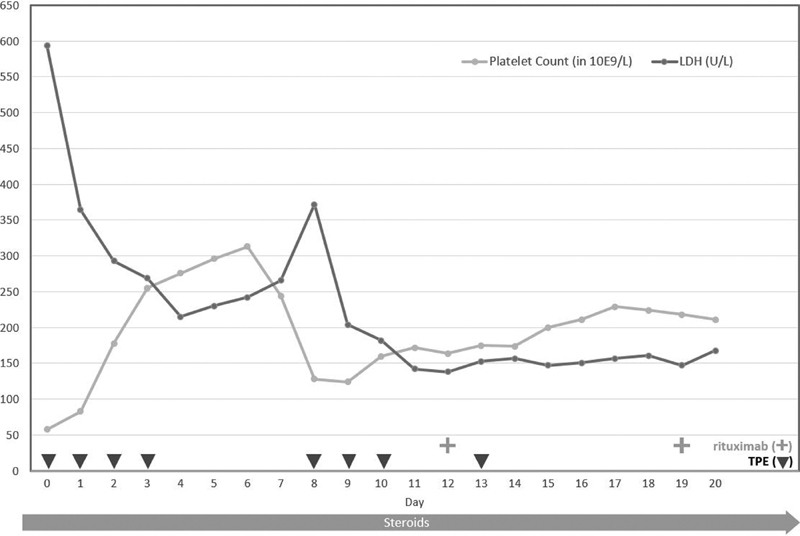
Laboratory trend versus treatments.

On the current visit (Day 0), the patient appeared jaundiced, diaphoretic and acutely unwell. He complained of lethargy, myalgias and anorexia for the past few days. Physical examination was also remarkable for irregularly irregular heartbeat, without hemodynamic instability. On history, he received his first dose COVID19 mRNA vaccine (Pfizer-Biontech) 7 days earlier. The patient denied any recent infections, or changes in his medications or overall health. Laboratory investigations revealed anemia (hemoglobin 7.2 g/dL), thrombocytopenia (58 × 10 ^9^ /L), few fragments on blood film and evidence of hemolysis (reticulocyte count elevated at 229, LDH elevated at 594 U/L, undetectable haptoglobin, and indirect hyperbilirubinemia - total bilirubin 61 and direct bilirubin 9 umol/L). This represented a significant change from his routine laboratory investigations performed a month earlier ( Table 1 ). EKG was consistent with a new finding of atrial fibrillation. In terms of acute complications, troponin high sensitivity was mildly elevated at 59ng/L (<18). Creatinine was also mildly elevated at 77umol/L (baseline 65) and urinalysis revealed 1+ protein and trace blood, but no ACR was done. He had no evidence of neurological complications on physical exam however, MRI brain revealed a new infarct in the left parietal lobe. The patient was hospitalized and treated with daily TPE and high dose steroids. Please note that local standard of care reserves Rituximab for patients with refractory TTP and caplacizumab is only available through a compassionate program for patients with refractory disease, severe end organ damage or religious objection to plasmatherapy. He received a total of 8 TPE treatments (Days 0, 1, 2, 3, 8, 9, 10 and 13). The patient had a rapid initial response and TPE was discontinued after 4 ^th^ treatment, having attained normalization of platelet counts and hemolytic markers (see Fig. 1 for laboratory trends). However, by Day 8, his platelet count started to decline, LDH to rise and haptoglobin became again undetectable. TPE was resumed and Rituximab was commenced (375mg/m ^2^ intravenously on Days 12, 19, 27 and 34). The patient was also started on apixaban 5 mg twice daily for stroke prophylaxis in the context of ongoing atrial fibrillation.The patient reached clinical remission by Day 10 and was discharged on Day 20. He made a complete recovery and was able to return to independent living. His ADAMTS13 activity remained undetectable, consistent with unresolved disease and high risk of imminent exacerbation. It eventually normalized and reached 47% after 1.5 months. Taking into account the possibility of triggering another relapse and expected poor immune response post Rituximab, decision was made to indefinitely postpone the administration of the second dose of vaccine.

**Table 1:** Select laboratory investigations before and after relapse

	1 month prior	Day 0 - Presentation	Day 20–Discharge date	Day 46–follow-up
Hemoglobin (g/dL)	14.2	7.2	9.9	13.5
Platelet count (E9/L)	183	58	211	227
Lactate Dehydrogenase (100–195U/L), U/L	Not available	594	168	219
ADAMTS13 activity, U/mL	Not available	<0.01	Not available	0.47

In summary, this case describes development of iTTP relapse and atrial fibrillation in an 84-year-old male, seven days after he received the first dose of COVID19 mRNA vaccine. The patient's clinical course was milder than his previous TTP episode, likely owing to an early diagnosis and treatment. This is only the second case report of immune TTP associated with COVID19 mRNA vaccine and adds to the overall literature on hematologic complications of COVID19 vaccines. In contrast, the previous case report by Sissa et al described an iTTP relapse within 6 days of the second dose of mRNA vaccine. In both cases, the patients recovered following TPE and immunosuppression. Considering the scale of COVID19 vaccination, immune TTP is likely a very rare complication of COVID19 vaccination. However, considering significant morbidity associated with TTP, these rare cases argue in favor of proactive laboratory and clinical monitoring of patients with iTTP within the first 2 weeks post vaccination to allow for early diagnosis and treatment.
